# Gender, Soft Skills, and Patient Experience in Online Physician Reviews: A Large-Scale Text Analysis

**DOI:** 10.2196/14455

**Published:** 2020-07-30

**Authors:** Zackary Dunivin, Lindsay Zadunayski, Ujjwal Baskota, Katie Siek, Jennifer Mankoff

**Affiliations:** 1 Indiana University Bloomington, IN United States; 2 Rensselaer Polytechnic Institute Troy, NY United States; 3 Jackson State University Jackson, MS United States; 4 University of Washington Seattle, WA United States

**Keywords:** reviews, physician-patient relationship, gender, soft-skills

## Abstract

**Background:**

Online physician reviews are an important source of information for prospective patients. In addition, they represent an untapped resource for studying the effects of gender on the doctor-patient relationship. Understanding gender differences in online reviews is important because it may impact the value of those reviews to patients. Documenting gender differences in patient experience may also help to improve the doctor-patient relationship. This is the first large-scale study of physician reviews to extensively investigate gender bias in online reviews or offer recommendations for improvements to online review systems to correct for gender bias and aid patients in selecting a physician.

**Objective:**

This study examines 154,305 reviews from across the United States for all medical specialties. Our analysis includes a qualitative and quantitative examination of review content and physician rating with regard to doctor and reviewer gender.

**Methods:**

A total of 154,305 reviews were sampled from Google Place reviews. Reviewer and doctor gender were inferred from names. Reviews were coded for overall patient experience (negative or positive) by collapsing a 5-star scale and coded for general categories (process, positive/negative soft skills), which were further subdivided into themes. Computational text processing methods were employed to apply this codebook to the entire data set, rendering it tractable to quantitative methods. Specifically, we estimated binary regression models to examine relationships between physician rating, patient experience themes, physician gender, and reviewer gender).

**Results:**

Female reviewers wrote 60% more reviews than men. Male reviewers were more likely to give negative reviews (odds ratio [OR] 1.15, 95% CI 1.10-1.19; *P*<.001). Reviews of female physicians were considerably more negative than those of male physicians (OR 1.99, 95% CI 1.94-2.14; *P*<.001). Soft skills were more likely to be mentioned in the reviews written by female reviewers and about female physicians. Negative reviews of female doctors were more likely to mention candor (OR 1.61, 95% CI 1.42-1.82; *P*<.001) and amicability (OR 1.63, 95% CI 1.47-1.90; *P*<.001). Disrespect was associated with both female physicians (OR 1.42, 95% CI 1.35-1.51; *P*<.001) and female reviewers (OR 1.27, 95% CI 1.19-1.35; *P*<.001). Female patients were less likely to report disrespect from female doctors than expected from the base ORs (OR 1.19, 95% CI 1.04-1.32; *P*=.008), but this effect overrode only the effect for female reviewers.

**Conclusions:**

This work reinforces findings in the extensive literature on gender differences and gender bias in patient-physician interaction. Its novel contribution lies in highlighting gender differences in online reviews. These reviews inform patients’ choice of doctor and thus affect both patients and physicians. The evidence of gender bias documented here suggests review sites may be improved by providing information about gender differences, controlling for gender when presenting composite ratings for physicians, and helping users write less biased reviews.

## Introduction

### Background

Physician review sites are relatively new and were initially greeted with concern by some in the medical community. In particular, some physicians were critical of the lack of transparency in composite statistics [1] and were concerned that online reviews could harm their careers [2]—perhaps unfairly [3,4]. Although ratings are generally high [2,3,5,6], negative ratings undoubtedly influence patient behavior [7] and impact doctors [1,8]. Some doctors have attempted to *gag* patients by contractually prohibiting them from writing online reviews [9].

Most studies of online physician reviews have focused on portals such as HealthGrades [10], RateMDs [11], Vitals [12], and Yelp [13]. Studies tend to have a small sample size, analyzing approximately 5400 reviews [6]. Many studies aim to understand the factors that influence quantitative physician ratings. The qualitative analysis of 712 reviews by López et al [5] established thematic categories that tended to appear in reviews. Paul et al [14] replicated and expanded this work with a natural language processing (NLP) approach, which they applied to the text of 50,000 online reviews downloaded from RateMDs [11]. Their novel joint topic–sentiment modeling approach found that certain textual accounts of interpersonal skills such as *rude*, *arrogant*, and *condescending* are strongly associated with negative reviews, and drew attention to the role of patient experience of bureaucratic process in reviews, noting that these experiences were often reflected in reviews. Wallace et al [15] expanded on the work by López et al [5] and Paul et al [14] by analyzing 60,000 reviews to identify relationships between overall rating, health outcomes, and cost of care. To date, the only study to investigate the relationship between physician reviews and gender is that by Nwachukwu et al [16] on surgeon quality in sports medicine. They found that communication style influenced the valence of ratings for top- and bottom-tier surgeons and that female surgeons typically had higher ratings [16]. These and other studies of online reviews endeavor to understand how clinical experiences influence patient satisfaction and health outcomes. However, they tend to overlook or minimize questions about whether online review data reflect real experiences of medical care. Reviews may not be representative of the public or reflect demographic variation in health care utilization; indeed, doctor reviews are typically written by educated, younger, affluent, and healthier people [5]. However, a study comparing ratings of over 3000 physicians with licensing data showed a clear relationship between doctor quality and ratings [3].

Little research has studied the impact of gender or other demographic factors on the content and ratings of online physician reviews. Although qualitative studies of doctor-patient relationships have considered both negative and positive experiences [5], including the impact of demographics [17-19], the nature of these studies makes it difficult to estimate the size or scope of gender and other demographic variation in online physician reviews. Furthermore, gender differences, in particular biased interpretations of clinical experiences based on gender stereotypes, may impact online review content, which in turn may negatively impact both patients and physicians and perpetuate false gender stereotypes. The large-scale systematic study we present here documents gender differences in patient reviews with respect to both patients and doctors and proposes improvements for online review systems that could help reduce these disparities, thus improving information quality.

### Gender and Health Care

Although we know little about gender in the context of online reviews, gender has been studied extensively in the social sciences for over half a century. Much of this work investigates the role of gender in medical care and health systems more generally.

Gender is a cultural construct that affects people’s expectations and actions [20]. In social contexts and practices [21], gender is *assessed* independently of one’s identity [22]. Thus, any name appearing in online text is likely to be interpreted in terms of the man/woman binary, which is reflected in the use of gender in the current doctor review literature.

The expectations of one’s behavior differ depending on one’s assessed gender. Indeed, leadership traits praised in men are penalized in women, while traditional *feminine* behavior is seen as ineffective [23]. When writing references, men are described with more standout and ability-based words and fewer *grindstone* words (eg, *hardworking*, *conscientious*) [24], and women are described with more communal words [25]. Even when all factors are controlled, people rated teachers differently on hard skills (eg, *promptness*, *fairness*) and soft skills depending on the gender portrayed by the instructor [26]. Thus, bias may influence review content even when performance is identical.

In health care, gender differences influence doctors’ communication with patients [27-29], doctor and patient trust [30], and even diagnosis error rates [27]. Female *doctors* are seen as partners and more involved in the patient-doctor relationship, whereas female *patients* are treated with more condescension [27,29,31], have their concerns dismissed [29] and credibility doubted [27]. Conversely, patient satisfaction is dependent on more caring communication styles for women than for men [32]. However, many studies documenting these trends are small in scale or have weak evidence [33]. One exception is a study of over 10,000 people experiencing long-term illness in Sweden, where women reported being blamed, interrupted, disbelieved, doubted, and regarded as stupid [19]. These gender differences are likely to impact review scores, as lower patient satisfaction is correlated with high physician dominance, which can manifest itself in gendered actions (eg, poor information sharing and use of medical jargon) [34]. In this study, we investigate how these gender biases are represented in online reviews, which affect patients, physicians, and people using the reviews.

### Study Design and Motivation

The goal of this study was to broaden and deepen our understanding of the impact of gender bias and other gender differences on online physician reviews. We leveraged reports of patient sentiments about their doctors through a large-scale analysis of online reviews. The Google Place review data analyzed here allowed us to identify patient and reviewer gender and characterize patient sentiment or experience in terms of both overall quality (a reviewer-entered Likert-type scale) and thematic content. Specifically, we formulated the following hypotheses (H):

- H1a—Physician ratings and physician gender: female physicians are more likely to receive negative reviews than male physicians.
- H1b—Physician ratings and reviewer gender: Female reviewers are more likely to report negative experiences with doctors.
- H2a—Soft skills and physician gender: Female physicians are more likely to receive criticism mentioning soft skills than male physicians.
- H2b—Soft skills and reviewer gender: Female reviewers are more likely to mention soft (interpersonal) skills in negative reviews.
- H3—Reviewer gender and physician gender: Female reviewers are more likely to report negative experiences with male doctors.

Hypotheses H1a and H1b relate to physician gender and reflect the findings of prior work on gender inequalities in reviewing in other fields [24-26]. Hypotheses H2a, H2b, and H3 are based on prior work documenting gender differences in clinical encounters [27,28].

Our approach comprised both qualitative and quantitative perspectives on review content that mutually informed one another throughout the research process. We collected and analyzed a corpus of 154,305 reviews of doctors that constitutes a large nationally representative sample of physician reviews across all medical subfields and clinical contexts. Our focus was specifically on characterizing the differences in experience quality and content as they relate to reviewer and doctor gender.

This study contributes to the larger body of work on the impact of gender on clinical interactions and provides insight into what patients value in their doctors. Furthermore, we add to the small but growing body of literature that seeks to develop a general understanding of online reviews and the systems that collect and display them. Our results must be interpreted with caution due to the unstructured and often short nature of the patient narratives in reviews, the relative crudeness of the NLP techniques employed (as compared with human interpretation), and selection biases introduced by nonrepresentative variation in demographic characteristics of reviews and the types of experiences that motivate patients to write reviews. Such selection biases are almost unknown although we make a novel contribution here.

Online reviews provide an opportunity to learn, at scale, about patients’ perceptions of their doctors. Our findings have direct implications for the design of review sites and the presentation of search results. We argued that there are a number of useful ways in which gender differences can be reflected and potentially corrected in the presentation of information on the internet.

## Methods

This study is a large-scale (*N*>10^5^) text analysis of reviews of US physicians in the form of social media trace data. Social media trace data have the advantage of feature richness, (often) wide availability, and relation to genuine human social behavior outside of an experimental or survey context. The analytic approach we pursued in this study followed a similar process to the hybrid ethnographic and NLP approach advocated by Nelson [35]. The methodological framework is a recursive process whereby qualitative text analysis (sometimes called *deep reading* or *content analysis*) informs computational feature extraction, which is then evaluated through further qualitative analysis. This refinement process continues until the patterns in the computationally derived features match the intuitions and examples accumulated through qualitative analysis fairly well. Ultimately, a quantitative analysis, in this case regression modeling, is applied to validate large-scale patterns in the data. The rest of this section details the specifics of this approach, the review sampling process, inference of reviewer and physician gender, and modeling of associations and interactions among the variables of interest.

### Physician Review Collection

To examine how gender influences patient experience at scale, we sought a representative sample of reviews of US physicians. As gender and other demographic variables are rare in social media, we sought data that contained physician and reviewer names, which we used as a proxy for gender.

### Review Collection Application Programming Interface

After exploring possible sources of physician reviews considering various application programming interface (API) features and use in prior work, we selected the Google *Places* API [36]. The API provides access to patient and physician names, which we leveraged to infer gender, as well as a broad range of areas and specialties. The Google My Business API has a 5-review limit for any particular doctor. Unfortunately, the API documentation does not provide information on how these reviews are selected. We can be fairly confident, however, that reviewer gender is not a factor. Thus, it is unlikely that this introduces bias into the sample with regard to the variables of interest. Furthermore, we took measures to ensure that our personal search histories did not influence review collection.

### Geographical Sampling

Reviews, physicians, and practices are likely to vary by location. Differences in locale, such as ruralness or urbanity, can influence health outcomes and care options, as can regional differences. For instance, in the United States, mortality rates of particular conditions have been shown to differ greatly from state to state [37]. Samples were taken across states from multiple regions of the United States using the Google Places API to control for the effect of locale. We steadily increased latitude and longitude intervals throughout each state with a 10,000-m radius to capture both urban and rural regions.

### Data Summary

Reviews returned by the Google My Business API were either for a *place* (such as a practice with multiple doctors) or a *physician*. The API provided additional *review-specific* data for each review: a 5-point Likert-type rating assigned by the reviewer to the doctor or practice, doctor name, reviewer name, the location of the practice, and the text content of the review. The reviewer and physician gender were determined automatically using third-party software described in the following section.

**Figure 1:**
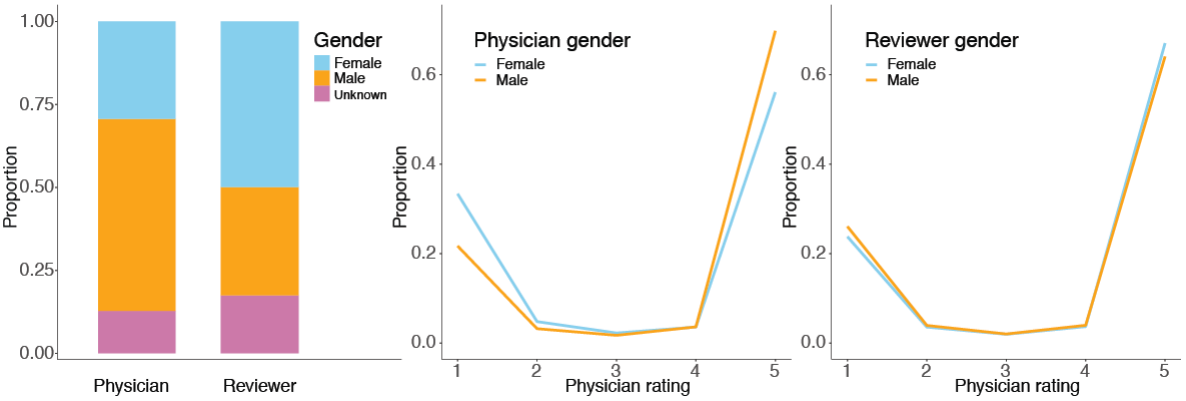
Left: Gender distribution in the complete data set (N=154,305). The unknown category represents clinics and names that were androgynous or unknown to the gender classifier. Middle: Distribution of physician ratings by physician gender (N=137,329). Right: Distribution of physician ratings by reviewer gender (N=129,985).

The collection strategy described in this section yielded 154,305 reviews of physicians across the United States. These reviews spanned 2007 to 2017. Doctor ratings were highly polarized, exhibiting a U-shaped distribution (Figure 1). Of the reviews collected, 46,605 were rated 1 star or 2 stars (*negative* reviews) and 107,700 were rated 4 stars or 5 stars (*positive* reviews). Another 3,208 reviews were rated 3 stars and were omitted from our analyses. We did not screen for particular specialties.

The mean length of positive reviews was 50 words, while the mean length of negative reviews was 100 word (both follow heavy-tailed distributions). The distribution of inferred gender for both doctors and reviewers is shown in Figure 1. Note that we expected to see a fairly high rate of unknown gender in these data because some reviews are for medical practices that include multiple physicians. In addition, some physicians’ gender could not be identified. Given the presence of nonperson entities among *physicians*, it is perhaps surprising that reviewers have a greater rate of gender ambiguity. This likely reflects typos and pseudonyms among reviewers, who have a weaker incentive to use the correct name. However, the high rate of gender detection suggests that this should not be a great concern, as almost all reviewers use *real* names, not screen names. We were less certain about the rate of pseudonymous users but proceeded on the assumption that even when pseudonyms are used, they accurately reflect the reviewer’s gender.

### Qualitative Coding

#### Sampling Strategy

To guide our quantitative analysis and support the validation of our approach, we additionally selected a small sample of reviews for hand coding. A total of 200 reviews were selected for hand coding using stratified sampling for a distribution of 60% negative (3/5 from each state) and 40% positive (2/5 from each state) reviews because our initial read-throughs indicated that negative verbiage was less prevalent than positive verbiage in our sample.

As this sample was relatively small in comparison with the number of total reviews collected, we used search terms intended to select for specialties that would help us focus on specific patient genders. We selected 50 reviews by mostly female reviewers using *maternal*, *fetal*, *fertility*, *natal* as search terms for clinic name and *maternity*, *fetal*, *miscarriage*, *trimester*, *fertility*, *natal*, *birth*, *pregnancy*, *delivery*, *baby*, midwife, *Ob/Gyn* in the review. We also selected 50 reviews by mostly male reviewers using *cancer*, *prostate* in the review itself, along with 100 reviews of both male and female urology reviewers. All of these reviews were manually assessed to ensure that they represented the assigned group. Although pregnancy and prostate cancer are not comparable medically, we chose them because they selected for patients by biological sex. They are the only common conditions that affect only one biological sex. This reduces uncertainty in our interpretation of the qualitative data.

We analyzed these reviews to construct a codebook (Multimedia Appendix 1) and develop an intuition for the patterns of thematic content and gendered interaction in physician reviews. Throughout this paper, we reflect on these patterns or illustrate particular situations by quoting largely from the reviews in this sample. The intuitions we developed through our qualitative and quantitative analyses have led us to conclude that situations reported in exemplar quotations generalize beyond the women’s health or urological contexts. Accordingly, our quantitative analysis and some follow-up quantitative investigations span the entire data set.

When we quote reviews, they will be cited with the following descriptors: physician gender (Male; Female; Unknown), star rating (1-star, 2-star,..., 5-star), doctor type (O=OBGYN; U=UROLOGY). For example, (Male; 5-star; O5654) is an OBGYN review of a male physician with a 5-star rating. Quotes are exemplary of many similar statements found in the reviews, with some synthesis and paraphrasing to support anonymization.

#### Codebook Construction

We developed a codebook to regulate our analysis of the reviews. These codes were divided into 2 parts: regular codes and context codes. Context codes relate to demographic information, doctor gender, or specialty, while regular codes reflect the content of the review, for example, *professional* or *rude*.

We began with codes identified by López et al [5]. We also used iterative open coding to identify common categories of statements in the data. After coding each of the qualitative samples in Text Analysis Markup System [38], we took each coded section and created an affinity diagram, grouping similar segments independent of the initial codebook to represent the content of the reviews most accurately. These were then grouped into overarching categories that could be used for the analysis of the full data set. The process resulted in 7 main thematic areas: *process*, *candor*, *trust*, *investment*, *amicability*, *indifference*, and *disrespect*. The themes represented 2 general categories: those pertaining to nursing and administrative *process* (process) and those pertaining to soft skills. The latter group was further subdivided into *positive* (candor, trust, investment, amicability) and *negative* (indifference, disrespect) soft skills. After developing the codebook, 3 authors coded 20 reviews to assess interrater reliability using Cohen kappa (Table 1) and then refined the categories to improve agreement.

**Table 1:** Themes that emerged in affinity diagramming and examples of the associated terms in the dictionaries used in the quantitative analysis.

Theme			Sample terms		Kappa^a^		Count		Accuracy^b^		Precision		Recall
**Positive soft skills**				0.61		113		0.77		0.74		0.90	
	Candor	Honest, explain, answer, direct		0.95		41		0.84		0.57		0.76	
	Trust	Support, safe, reassure, comfort		0.98		27		0.42		0.41		0.63	
	Investment	Respect, care, compassion, listen		0.93		65		0.42		0.60		0.74	
	Amicability	Warm, friendly, personable, funny		0.94		31		0.82		0.46		0.84	
**Negative soft skills**				0.53		25		0.87		0.44		0.16	
	Indifference	Cold, dismiss, ignored, abandoned		0.74		16		0.87		0.50		0.16	
	Disrespect	Rude, harass, condescending, arrogant		0.40		25		0.92		0.50		0.44	
Process			Cost, nurse, staff, wait		0.83		115		0.87		0.84		0.96

^a^Kappa represents interrater agreement (on 20 reviews).

^b^Accuracy, precision, and recall, respectively, on a random sample (*N*=100) of 200 total reviews. A review is labeled as pertaining to a theme if at least one of the words in the theme in dictionary is presented in the review. Note the infrequency of negative soft skills (16 and 25 for indifference and disrespect, respectively), contributing to low precision and recall.

### Computational Feature Extraction

#### Gender Detection

A third-party Python library [39] was used to infer the probable gender of physicians and reviewers based on their name. Although not everyone identifies within the female-male gender binary [22], currently, gender is typically assessed and reacted to with respect to this binary [21], so a binarized gender of reviewers and physicians was extracted using the names provided. Although a binary definition of gender does not capture the spectrum of gender and gender relations, capturing a more complex understanding of gender is infeasible given the scope of our data and the lack of identifying information for reviewers and doctors beyond name.

To verify the accuracy of the gender inference procedure, we took a random sample of 200 reviews and compared automatically inferred gender with our human-coded gender determinations informed by close reading of the reviews informed by name and gender pronouns. Automated physician gender inference was 98% accurate. The accuracy of reviewer gender was not examined because the only available measure was the reviewer’s name.

The distribution of genders is shown in Figure 1 (left). Gender could not be inferred for 12.8% of physicians and 17.5% of patients. A logistic regression model (not shown) estimated with high confidence that female physicians are 1.41 times more likely to be reviewed by female patients (*P*<.001). A second model (also not shown) indicates that physicians are 4.36 times more likely to be reviewed by patients of the same gender (*P*<.001). These figures do not necessarily represent the actual gender distribution of patients *seen* by doctors, as there may be selection bias for or against intragender reviews.

#### Thematic Content of Reviews

Informed by the qualitative analysis, dictionaries were developed relating to the 7 themes (*process*, *candor*, *trust*, *investment*, *amicability*, *indifference*, and *disrespect*) identified in the qualitative coding. Review text was stemmed using Porter stemmer and tagged with a binary label for each theme if the review mentioned a word in the theme’s dictionary. Working separately, we coded 200 reviews to assess the ability of the codebook to identify each of the themes. Table 1 presents summaries of the themes, their kappa statistics, and the performance of the binary variables when applied as a single-feature classifier against a random test set of 100 hand-coded reviews that were not used to inform the codebook. Tables 2 and 3 display the frequencies with which the terms appear in the corpus and the proportion of reviews for each combination of gender/physician rating for physician gender and patient gender separately.

**Table 2:** Prevalence of themes by physician gender.

Theme			Doctors (female, n=36,847; male, n=74,189)^a,b^						
			Female (negative; n=13,874), n (%)^c^		Female (positive; n=22,973), n (%)^c^		Male (negative; n=17,906), n (%)^c^		Male (positive; n=56,283), n (%)^c^
**Positive soft skills**			7403 (53.36)		16,984 (73.93)		7530 (42.05)		37,993, (67.50)
	Candor	2756 (19.86)		3531 (15.37)		2227 (12.44)		8431 (14.98)	
	Trust	754 (5.43)		2725 (11.86)		745 (4.16)		5566 (9.89)	
	Investment	4724 (34.05)		12,266 (53.39)		4830 (26.97)		26,032 (46.25)	
	Amicability	1928 (13.90)		7481 (32.56)		1621 (9.05)		16,047 (28.51)	
**Negative soft skills**			4629 (33.36)		488 (2.12)		4505 (25.16)		721 (1.28)
	Indifference	868 (6.26)		163 (0.71)		836 (4.67)		298 (0.53)	
	Disrespect	4112 (29.64)		343 (1.49)		3942 (22.01)		442 (0.79)	
Process			9330 (67.25)		10,099 (43.96)		9981 (55.74)		23,837 (42.35)

^a^Many reviews contain multiple themes, so the overall rows (bold) have smaller numbers than the sum of themes would indicate. This table includes only those reviews for which a gender was assigned (n=111,036).

^b^The physician rating is denoted as negative/positive.

^c^Percentages represent the proportion of reviews containing the theme for that particular gender/rating combination.

**Table 3:** Prevalence of themes by reviewer gender.

Theme			Reviewers (female, n=67,857; male, n=43,179)^a,b^						
			Female (negative; n=18,780), n (%)^c^		Female (positive; n=49,077), n (%)^c^		Male (negative; n=13,000), n (%)^c^		Male (positive; n=30,179), n (%)^c^
**Positive soft skills**			9102 (48.47)		35,334 (72.00)		5831 (44.85)		19,643 (65.09)
	Candor	3104 (16.52)		7815 (15.92)		1879 (14.45)		4147 (13.74)	
	Trust	906 (4.82)		5636 (11.48)		593 (4.56)		2655 (8.80)	
	Investment	5834 (31.06)		24,994 (50.93)		3720 (28.62)		13,304 (44.08)	
	Amicability	2231 (11.88)		15,630 (31.85)		1318 (10.14)		7898 (26.17)	
**Negative soft skills**			5868 (31.25)		790 (1.61)		3266 (25.12)		419 (1.39)
	Indifference	1101 (5.86)		305 (0.62)		603 (4.64)		156 (0.52)	
	Disrespect	5178 (27.57)		513 (1.05)		2876 (22.12)		272 (0.90)	
Process			11,469 (61.07)		21,728 (44.27)		7842 (60.32)		12,208 (40.45)

^a^Many reviews contain multiple themes, so the overall rows (bold) have smaller numbers than the sum of themes would indicate. This table includes only those reviews for which a gender was assigned (N=111,036).

^b^The physician rating is denoted as negative/positive.

^c^Percentages represent the proportion of reviews containing the theme for that particular gender/rating combination.

Dictionary-based text analysis is crude in that it cannot determine valence, that is, the dictionary approach cannot distinguish between the phrase “Dr. X listens,” for example, “Great bedside manner. She was kind and listened to everything I had to say” (Female; 5-star; 15180), and the many variants of its negation, for example, “I never felt like she truly listened” (Female; 1-star; 25068). Positive soft skills are more likely to be negated than negative ones, largely because double negatives are far less common than single negatives in English. This is borne out by the associations between each theme and *negative reviews* in the set of models summarized in Table 4. Determining valence is further complicated by constructs that contradict a negative, such as “If you want a doctor who knows what’s best after not listening to you for 5 minutes, don’t see him. This quality of care is almost impossible to find” (Male; 5-star; 40558). As reviewers have assigned a general valence to their experience, we leveraged physician ratings to distinguish positive from negative sentiment. However, this applies only in analyses where positive reviews are considered in isolation from negative reviews and vice versa. In the quantitative analyses below, we typically controlled for interactions between gender and physician rating. Therefore, when considering a soft skill in the context of reviews with ratings of the opposite valence, the soft skill should be interpreted as the negation of that interpersonal trait. For instance, when *amicability* appears in a negative review, the reader should interpret this as the *absence* of amicability, whereas *disrespect* in the same review should be regarded as the *presence* of disrespect.

**Table 4:** Logistic regression on the presence of a theme in review (n=106,325).

Model^a^		Intercept	Doctor_F_^b^	Reviewer_F_^b^	Rating_Neg_	Doctor_F_×Rating_Neg_	Reviewer_F_×Rating_Neg_	Reviewer_F_ ×Reviewer_F_
**No interactions**								
	Candor	−1.92^c^	0.15^c^	0.16^c^	−0.10^c^	—^d^	—	—
	Trust	−2.40^c^	0.19^c^	0.27^c^	−1.01^c^	—	—	—
	Investment	−0.40^c^	0.27^c^	0.21^c^	−0.88^c^	—	—	—
	Amicability	−1.57^c^	0.25^c^	0.23^c^	−1.20^c^	—	—	—
	Indifference	−5.26^c^	0.29^c^	0.18^c^	2.19^c^	—	—	—
	Disrespect	−5.30^c^	0.35^c^	0.24^c^	3.39^c^	—	—	—
**Doctor**_F_×**Rating**_Neg_								
	Candor	−1.88^c^	0.01	0.16^c^	−0.31^c^	0.47^c^	—	—
	Trust	−2.39^c^	0.18^c^	0.27^c^	−1.03^c^	0.04	—	—
	Investment	−0.40^c^	0.26^c^	0.21^c^	−0.89^c^	0.01	—	—
	Amicability	−1.56^c^	0.20^c^	0.23^c^	−1.34^c^	0.29^c^	—	—
**Reviewer**_F_×**Rating**_Neg_								
	Candor	−1.92^c^	0.15^c^	0.16^c^	−0.10^c^	—	−0.00	—
	Trust	−2.42^c^	0.19^c^	0.31^c^	−0.86^c^	—	−0.23^c^	—
	Investment	−0.42^c^	0.27^c^	0.25^c^	−0.80^c^	—	−0.14^c^	—
	Amicability	−1.58^c^	0.25^c^	0.25^c^	−1.12^c^	—	−0.13^e^	—
**Doctor**_F_×**Reviewer**_F_								
	Disrespect	−5.34^c^	0.46^c^	0.31^c^	3.39^c^	—	—	−0.16^e^

^a^Rows represent distinct logit models for each of the 7 themes. Each cell reports the log-likelihood that a variable is associated with the given theme. Sentences containing terms related to the process have been removed from the reviews.

^b^Female=1, male=0.

^c^*P*<.001.

^d^Missing value indicates that no coefficient was estimated for the given endogenous variable.

^e^*P*<.01.

### Quantitative Analysis

A total of 3 sets of logistic regression models were fitted to these data. Reviews for which either reviewer or physician gender could not be identified were removed from the analysis in all of the models presented in the Results section. This reduced the data set by 28% from 154,305 reviews to 111,036.

The set of models we present investigated the association between negative reviews and reviewer/doctor gender. The second set comprised models examining the likelihood that a review mentions a soft skill. As our primary variables of interest are binary, and we are interested in interactions among those binary variables, the interpretation of the logistic regression variables is complicated. All interaction terms disrupt the interpretation of their component variables, but this interpretation is even more difficult when estimating all pairwise interactions of a set of variables (in this case, 3). Effectively, this decomposes each main effect coefficient into different components, which must be carefully interpreted and summed to construct odds ratios (ORs) for various conditions. We present one set of regressions on each review theme for each interaction rather than estimating all 3 interactions in a single model for readability. Through these sets, we tested *physician gender*, *reviewer gender*, and *physician rating* for pairwise interactions. The intercept and noninteracting effects are only marginally altered between these models, if at all. An additional set of models estimates the main effects of each dependent variable. When reporting these results, models are grouped by these model classes rather than by the dependent variable, as the relation between the independent variables takes priority over the particulars of the review themes. Finally, a pair of models estimates how administrative process correlates with gender, physician ratings, and soft skills.

## Results

This section discusses the statistical models fit to the review data. The report and discussion of these results is supplemented with excerpts of real reviews examined in the qualitative component of this study. We present these reviews to illustrate and contextualize the quantitative findings and the computational method of feature extraction.

### Physician Ratings and Gender

Owing to the possibility that reviews could be influenced by clinical processes outside of physician control, we first fit a pair of models with and without mentions of bureaucratic *process*. The logit models summarized in Table 5 indicate that physician ratings are extensively influenced by gender, irrespective of mentions of *process*. Four models examine the correlation between sets of independent variables and the probability of a *negative review*. The *A* models were fit on all reviews for which we were able to infer both doctor and reviewer gender (*N*=111,036). In the data set on which we regressed the *B* models, we stringently controlled for mentions of *process*, which refer not to the physician but instead to the clinical aspects beyond the patient-physician relationship. The data set for the *B* models is the result of filtering sentences that mention terms associated with *process* from the reviews and then removing any reviews that were left without text. This process yielded a slightly smaller data set (*N*=106,325). Models 1A and 1B present a base model that includes only physician and reviewer gender and an interaction term. Models 2A and 2B control for soft skills, with Model 2A controlling for *process*. The overlapping coefficient estimates do not differ substantially between the 4 models, suggesting that mentions of *process* do not substantially alter the correlations captured by the variables of interest in these data. However, we conservatively controlled for them in the remainder of this section, except in models that consider *process* explicitly.

**Table 5:** Logistic regression on rating negative (A: n=111,036; B: n=106,325). Models 1 and 2 differ in the inclusion of review content themes. The B variants show the effects of filtering sentences mentioning process from each review.

Variable	Model 1A	Model 1B (no process)	Model 2A	Model 2B (no process)	*P* value
Intercept	−1.05	−1.08	−1.12	−0.80	<.001
Doctor_F_^a^	0.67	0.69	0.63	0.71	<.001
Reviewer_F_^a^	−0.16	−0.14	−0.14	−0.11	<.001
Doctor_F_×Reviewer_F_	−0.03	−0.05	−0.02	−0.03	NS^b^
Candor	N/A^c^	N/A	0.06	0.05	<.05
Trust	N/A	N/A	−0.95	−0.92	<.001
Investment	N/A	N/A	−0.89	−0.90	<.001
Amicability	N/A	N/A	−1.49	−1.18	<.001
Indifference	N/A	N/A	2.35	2.28	<.001
Disrespect	N/A	N/A	3.42	3.45	<.001
Process	N/A	N/A	0.84	N/A	<.001

^a^Female=1, male=0.

^b^NS: not significant.

^c^Not applicable.

Although the dictionaries that contain terms related to review themes were developed in conjunction with our qualitative analysis and were thus thoroughly vetted, there remained concerns that these term lists do not adequately capture the themes they purportedly represent. As discussed in the Methods section, dictionary-based or *bag of words* (presence or absence of terms) approaches to natural language understanding often struggle to overcome or capture nuance in word use, notably suffering an inability to distinguish positive use from negation. We fit 2 models (2A and 2B) to verify that the soft skill dictionaries are correlated with negative reviews, as expected. The coefficients for the themes indicate that our dictionaries capture the basic tendency we anticipated: 3 of 4 positive soft skills, *trust*, *investment*, and *amicability*, are correlated with positive reviews (negative coefficients; *P*<.001), and 2 negative soft skills, *indifference* and *disrespect*, are correlated with negative reviews (*P*<.001). Notably, *candor* is not associated with either positive or negative reviews. This suggests the possibility that *candor* was mischaracterized by its dictionary. However, the intercoder agreement and classifier performance in Table 1 imply that the dictionary for *candor* captures the theme equally well as the other positive soft skills. Rather, it seems that *candor* was misclassified as a *positive* soft skill and, as defined by its dictionary, is perhaps better understood as a *neutral* soft skill, appearing equally in positive and negative reviews. The models estimate large absolute effects for the three other positive soft skills. A negative soft skill, *indifference*, fits a considerably greater effect than any positive soft skill, and *disrespect* fits an even greater effect. This is consistent with the descriptive statistics in Tables 2 and 3, which suggest that although positive soft skills are more strongly associated with positive reviews, they also appear often in negative reviews. Negative soft skills by contrast occur overwhelmingly in negative reviews. Generally, these findings further suggest that our dictionaries accurately represent the themes they attempt to capture.

#### H1a: Female Physicians Are More Likely to Receive Negative Reviews Than Male Physicians

She was harsh and short. I always felt rushed and uncomfortable... it was like she was just making sure she did what was required. No sympathy at all.

Female; 1-star review; O6198

The logit models on physician ratings (Table 5) indicate that female doctors are considerably more likely to receive negative reviews. Model 1B, which includes only physician and reviewer gender and an interaction term, estimates that female physicians’ reviews are 2.00 (95% CI 1.90-2.10) times as likely to be negative than the reviews of male physicians (log OR 0.69, 95% CI 0.65-0.74; *P*<.001). Model 2B, which controls for mentions of soft skills, estimates a slightly larger coefficient.

#### H1b: Female Reviewers Are More Likely to Report Negative Experiences With Doctors

Contrary to our hypothesis, Model 1B estimates that men write negative reviews at 1.15 (95% CI 1.08-1.16) times the rate of women (*P*<.001). Controlling for review content themes (Model 2B) fit a slightly smaller estimate. There was no interaction between physician gender and reviewer gender, indicating that female patients are no more likely to give a doctor of a particular gender a negative review than men are.

#### Patient Experience, Physician Ratings, and Gender

Several batteries of logistic regression models were fit to investigate how specific aspects of the patient experience (review themes) interact with gender and overall patient experience (physician rating). The coefficients estimated by these models are listed in Table 4. As described in the Methods section, we fit separate models for each of the interaction terms, as the interpretation of multiple interaction terms is complicated, and separating them into distinct models does not significantly alter the results. We fit models that interact for gender and physician ratings for positive soft skills only, as negative soft skills are almost exclusively found in negative reviews. Furthermore, we report the gender×gender interaction model for disrespect only, as no other model estimated a significant interaction.

As comments about *process* may be wrongly ascribed to a doctor’s soft skills, the model for each theme controls for mentions of *process*. We found that *process* was significantly associated with all soft skills (*P*<.001), including a strong correlation with *amicability* and *disrespect* (see Table 6 and *Process and Gender* section for a more detailed treatment of this model). The model estimates that *process* is 2.73 (95% CI 2.65-2.86) times as likely to co-occur with *amicability* and 2.02 (95% CI 1.92-2.13) times as likely to co-occur with *disrespect*. This is unsurprising given our qualitative investigation, which found that the reviewers commonly commented on the friendliness or rudeness of the staff. For instance, when positive reviews mentioned both *process* and *disrespect*, it almost always contrasted a positive experience with a physician with a negative process experience. Reviewers seemed to be fairly capable of separating feelings about bureaucratic process from their experience with a physician, setting their dissatisfaction with, for example, staff, insurance, or booking aside when assigning a rating to a doctor who otherwise provided a good clinical experience.

### Physician Ratings and Soft Skills

In our qualitative analysis, we observed that reviews mentioning positive soft skills were primarily associated with high scores for male and female doctors. Reviewers wrote positively about physicians who were candid and direct, “Ladies this doctor listens and responds with respect, she does not talk down to you either” (Female; 5-star; U940); who were trustworthy and supportive, “The delivery would’ve been terrifying without him” (Male; 4-star; O7390); invested, “She asks questions and listens. She makes me feel like I am important” (Female; 5-star; U933); and amicable, for example, “She is an amazing doctor. Kind, caring, empathetic, warm, knowledgeable, quick thinking, funny, and honest” (Female; 5-star; O679). As stated in the discussion of the logit models in Table 5, positive soft skills are more likely to appear in positive reviews (*P*<.001). These models also estimate large effects for positive soft skills except *candor*, being at least 2.4 times as likely to appear in positive reviews than negative ones.

We also coded for 2 negative soft skills, *indifference* and *disrespect*. *Indifference* was relatively rare, appearing in only 2% of the reviews. *Disrespect* was more common, occurring in 8% of all reviews. Unlike positive soft skills, which appeared in both positive and negative reviews, negative soft skills were almost exclusively found in negative reviews. When they did appear in positive reviews, it almost always referred to bureaucratic process, not the physician. Typical reviewer comments coded for negative soft skills relate experiences with doctors who lack courtesy, patience, and warmth toward their patients, for example, “I could not believe how condescending and snippy she was!” (Female; 1-star; O8376); “He was very rude, condescending, arrogant, and appeared angry” (Male; 1-star; U1122). Reviewers also described feeling ignored, “I felt passed around and ignored” (Female; 1-star; O10061), or that their concerns were dismissed, “...brushed it off” (Female; 1-star; O10100) and “I was in tears because he was too stubborn to listen” (Male; 1-star; U1047). Other complaints included ignoring patients’ understanding of their own medical condition and lack of inclusion in decision-making. As reviewers mentioned, “The doctor does not listen to you and forces his opinion down your throat without considering your view. Do not visit here” (Male; 1-star; U398) and “He did not want to listen to anything I had to say and he definitely didn’t want me getting a second opinion. He got defensive and standoffish at the mention of any other opinion which says SHADY all over it” (Male; 1-star; U198).

The logit models for negative soft skills indicate that negative soft skills are far more likely to appear in negative reviews than positive ones. The log-likelihood coefficients are considerably stronger for negative than for positive soft skills. This is because positive soft skills can be negated to note the absence of a positive quality, whereas negative soft skills are rarely negated to indicate a positive quality.

#### H2a: Female Physicians Are More Likely to Receive Criticism Mentioning Soft Skills Than Male Physicians

I feel 100 percent comfortable telling her anything because I know she holds no judgment and treats everybody with fairness and kindness.

Female; 5-star review; O8982

The coefficients in the *Doctor*_F_ × *Rating*_Neg_ indicate that positive soft skills are more likely to occur in reviews of female physicians. In positive reviews, *trust*, *investment*, and *amicability* were more strongly associated with the reviews of female doctors than those of male doctors (*P*<.001). Trust and investment show no significant interaction between physician gender and physician rating, indicating that all reviews of female physicians are more likely to mention *trust* and *investment* than those of men. The models estimate that *trust* occurs 1.20 (95% CI 1.15-1.26) times and *investment* 1.31 (95% CI 1.27-1.34) times as often in reviews of female physicians than in those of male physicians. *Amicability* is estimated to have a significant effect on positive reviews and an additional amplifying effect in negative reviews. Positive reviews of female physicians reported *amicability* more often than those of male physicians (log OR 0.20, 95% CI 0.17-0.24; *P*<.001). To calculate the probability of mentions of *amicability* in female doctors’ negative reviews, we summed the *base* (that of positive reviews) log OR (0.20) with the log OR (0.29, 95% CI 0.19-0.38; *P*<.001) from the interaction term. The model estimates that *amicability* is much more likely to be mentioned in negative reviews of female physicians than in reviews of male physicians (log OR 0.49, 95% CI 0.36-0.62). Similarly, *candor* is much more likely to appear in negative reviews of female physicians (log OR 0.48, 95% CI 0.35-0.60; *P*<.001), although it is equally likely to appear in male and female physicians’ positive reviews.

Negative soft skills are more easily interpreted than positive ones, as they are less likely to be negated and thus occur predominately with negative valence. As discussed earlier, this is supported by the stronger associations of negative soft skills with negative reviews than those of positive soft skills and positive reviews. Qualitative analysis of reviews indicated that *disrespect*, when it occurs in positive reviews, usually refers to *process*. However, occasionally, reviewers will contradict or justify negative soft skills when referring to positive experiences with physicians, for example, “Some may misinterpret her candor as rudeness, but I appreciate that about her - she always gets right to the point” (Male; 5-star; 951) and “He can seem somewhat arrogant, but I’ve been seeing him for a while now, and he really knows his stuff and takes his patients very seriously. He has a right to think highly of himself!” (Male; 5-star; 7181).

Given their overwhelmingly negative valence, it is sufficient to model negative soft skills without interactions for gender and overall review quality (the *No Interactions* section of Table 4). However, it is important to control for physician ratings given the much higher rate of negative reviews in women’s reviews. Both *indifference* (log OR 0.29, 95% CI 0.20-0.37; *P*<.001) and *disrespect* (log OR 0.35, 95% CI 0.30-0.41; *P*<.001) are more likely to be mentioned in reviews of female physicians than in reviews of male physicians.

#### H2a: Female Reviewers Are More Likely to Mention Soft Skills in Negative Reviews

Our qualitative analysis did not reveal consistent patterns of association between mentions of soft skills and reviewer gender. As this investigation was limited to a small sample of reviews, we expected the quantitative results to yield patterns consistent with observations of gender differences in patients’ clinical experience reported in the literature. The logit models of soft skills offer 2 advantages in detecting gender bias in clinical settings. First, the high volume of observations may detect a pattern that was too rare to emerge from the qualitative analysis. Second, it may be that gender bias is not explicitly identified in most reviews but rather emerges when looking at the reviews in aggregate. The logistic models of soft skills demonstrate several associations between reviewer gender and soft skills, including interesting interaction effects.

The *Doctor*_F_ × *Rating*_Neg_ models in Table 4 find that all positive soft skills are more likely to occur in reviews written by women than those written by men (*P*<.001). *Candor* is roughly 1.17 (95% CI 1.13-1.21) times more likely to appear in all reviews written by women. The other 3 positive soft skills demonstrate a higher rate among women in positive reviews and a compensatory effect in women’s negative reviews. However, this effect merely dampens the greater probability of occurring in reviews written by women, not equalizing it. In the models that did not fit coefficients for gender and rating interactions (*No Interactions*), both *indifference* (log OR 0.18, 95% CI 0.09-0.27; *P*<.001) and *disrespect* (log OR 0.24 95% CI 0.18-0.30; *P*<.001) were more likely to appear in reviews written by women.

#### H3: Female Reviewers Are More Likely to Report Negative Experiences With Male Doctors

I have hunted for a female Urologist for several years. I was dealing with a male doctor who kept blowing off my concerns as a woman and telling me what women think they feel or know.

Female; 5-star review; U940

When women mentioned soft skills, they occasionally related difficulties with their doctor to physician gender. However, female reviewers rarely attributed poor treatment to their womanhood or to male physicians treating women poorly. It was also rare that women commented on the absence of bias in settings where they might have expected it, for example, “While he treats women and men, I think his sensitivity makes him especially good with women” (Male; 5-star; U1081).

To examine whether women or men report differential treatment depending on the gender of their doctor, each model tested an interaction effect between physician gender and patient gender. Only *disrespect* produced a highly significant gender×gender interaction. The model for *disrespect* estimates log OR of −0.16 (95% CI −0.28-−0.04; *P*=.008) for women who review female doctors. The reader may be inclined to interpret the negative coefficient for gender×gender interaction as evidence that women are less likely to report *disrespect* when seeing a female physician. This is true, but it must be qualified when we ask the question, *less likely relative to what*?

The odds ratio for the gender×g interaction indicates that the discrepancy in reports of disrespect between women and men is not so great when seeing a female doctor as we would have expected given the difference between women and men when seeing a male doctor. Female reviewers would seem to benefit from seeing female doctors, as we cannot reject the null that men and women report *disrespect* from a female doctor at equal probability. The *Doctor*_F_ × *Reviewer*_F_ model of *disrespect* estimates women to be 1.37 (95% CI 1.26-1.49) times as likely (log OR 0.31, 95% CI 0.23-0.40; *P*<.001) as men to report *disrespect* when seeing a male doctor (the *Reviewer*_F_ column). Given the interaction term, this probability represents the OR that a woman (compared with a man) reports *disrespect* from a *male doctor*. Summing this *base* probability with the interaction coefficient (log OR −0.16) estimates that female reviewers are 1.16 (95% CI −0.05-0.36) times (log OR 0.15) more likely to report *disrespect* when seen by a female doctor than a *man* seeing a female doctor. As the 95% confidence interval overlaps 0; we cannot reject the null hypothesis, that men and women report disrespect with the same probability when seeing women doctors. Similarly, when a man reviews a female physician, he is 1.58 (95% CI 1.43-1.74; log OR 0.46, 95% CI 0.36-0.55) times more likely to associate her with *disrespect* than he would a male doctor. The compensatory effect of the gender×gender interaction coefficient diminishes, but does not dissolve, the probability that a female doctor is reported to be disrespectful. When reviewed by female patients, female doctors are 1.35 (95% CI 1.08-1.67) times more likely to be associated with *disrespect* than a male doctor.

### Process and Gender

Although we made no predictions about administrative *process*, it is worth noting several patterns that emerged from the logistic models on mentions of *process*. We fit 2 models of *process* reported in Table 6 in the reviews for which gender could be inferred and which did not filter out mentions of *process* (*N*=111,036). Model 1 parallels the models of themes in Table 4 and accordingly reports interaction effects as separate models. Model 2 estimates correlations between each theme and *process*, controlling for rating and gender.

**Table 6:** Logistic regression on the presence of process in review (N=111,036).

Variable	Model 1			Model 2	
	Doctor_F_^a^×Rating_Neg_	Reviewer_F_^a^×Rating_Neg_	Doctor_F_×Reviewer_F_		Themes
Intercept	−0.37^b^	−0.41^b^	−0.43^b^		−0.84^b^
Doctor_F_	0.06^b^	0.20^b^	0.19^b^		0.12^b^
Reviewer_F_	0.11^b^	0.11^b^	0.14^b^		0.04^c^
Rating_Neg_	0.54^b^	0.70^b^	0.78^b^		0.83^b^
Doctor_F_ × Rating_Neg_	0.42^b^	—^d^	—		—
Reviewer_F_ × Rating_Neg_	—	−0.02	—		—
Doctor_F_ × Reviewer_F_	—	—	−0.13^b^		—
Candor	—	—	—		0.26^b^
Trust	—	—	—		0.34^b^
Investment	—	—	—		0.22^b^
Amicability	—	—	—		1.01^b^
Indifference	—	—	—		0.00
Disrespect	—	—	—		0.70^b^

^a^Female=1, male=0.

^b^*P*<.001.

^c^*P*<.01.

^d^Missing value indicate that no coefficient was estimated for the given endogenous variable.

*Process* is much more likely to be mentioned in negative reviews (*P*<.001). This is consistent across all models. This may be because when process is smooth, it is more likely to go unnoticed, whereas poor experiences with process are more likely to color the overall experience. Parallel to the trend observed in soft skills, *process* more often occurs in reviews written by and about women (*P*<.001). The *Doctor*_F_ × *Rating*_Neg_ model estimates that negative reviews of female physicians are 2.61 (95% CI 2.41-2.88) times as likely to mention process than negative reviews of male physicians (log OR 0.96, 95% CI 0.88-1.06; *P*<.001). By contrast, female and male reviewers mention process in negative reviews at equal rates. Women who see female doctors are less likely to mention *process*, which produces an equalizing effect that offsets the greater rate of reports of *process* for both female reviewers and physicians.

We also examined the association of *process* with soft skills. Model 2 demonstrates a positive correlation between mentions of *process* and soft skills. The correlations with *amicability* and *disrespect* are sizable (log OR 1.01, 95% CI 0.98-1.04 and 0.70, 95% CI 0.65-0.76, respectively; *P*<.001), indicating that patients value the ease of interpersonal interactions with staff, that is, whether they are *friendly* or *rude*, and likely interpret bureaucratic competence through the framework of how *nice* the staff are. In our qualitative analysis, we found that positive reviews expressing *disrespect* overwhelmingly do so with regard to *process*, indicating that reviewers are able to separate relationships with their doctor and the overall clinical experience as reflected in the final review. This compartmentalization is well illustrated by a patient who reported a positive experience with a doctor but faced problems with poor administration such that the patient ultimately severed their relationship with the clinic:

Terrific bedside manner!! Really dedicates time to patients and will even follow up by phone. The staff are rude and incompetent though. They repeatedly failed to file paperwork with my insurance. I got fed up with it and had to find a new doctor.

Unknown; 5-star review; 3686

## Discussion

### Interpreting These Results

Our results provide compelling evidence for a number of effects of gender on patient experience, as reported in physician reviews. These findings may be interpreted through 2 distinct frames. First, a *patient experience* frame attempts to interpret gender dynamics in the context of the patient-physician relationship. This frame should be familiar to readers versed in the literature on gender and clinical experience. The second frame, the *online review system* frame, seeks not to understand or improve the clinical aspects of health care but rather considers how gender differences may subvert or be leveraged to improve reviews as a valuable public resource that informs decisions about care-seeking.

The following discussion of the results in context of the hypotheses of this study assumes the *patient experience* perspective. The Summary and Recommendations section, however, largely reflects the *online review system* perspective, which is less concerned with controlled statistical inference than it is how the descriptive statistical patterns in Tables 2 and 3 might affect public perception of physician quality and how we might design online review systems to improve physician-patient matching and offset bias.

### H1a: Physician Ratings and Physician Gender

We hypothesized that female physicians would be more likely to receive lower ratings. This hypothesis was supported by our study. These data indicate that there is considerable reviewer bias against female physicians. This is consistent with well-documented patterns of bias against women in other fields, notably when reviewing instructor performance in a controlled online classroom [26].

### H1b: Reviewer Gender and Rating

We hypothesized that female patients would report overall worse clinical experiences than men. The results here support the opposite hypothesis. We found that men are slightly more likely to report negative experiences than women.

There are several valid interpretations of these findings. Men may receive worse care than women, as captured by patient experiences (rather than health outcomes). Alternatively, it could be that men have higher expectations for care than women or are less competent at navigating the clinical setting. Both are plausible given that men less frequently utilize health services [40,41]. Finally, we might attribute the discrepancy to women’s greater propensity for agreeableness [42,43], and for forgiveness [44] and compromise [45] in interpersonal conflict. These interpretations are not mutually exclusive, and further research is warranted to account for this trend.

### H2a: Soft Skills and Physician Gender

We hypothesized that reviews of female physicians would be more likely to critique their soft skills. Our results indicate that this is true of all soft skills. All soft skills were more often mentioned in the reviews of female physicians. In negative reviews of female physicians, reviewers were considerably more likely to mention *candor* and *amicability*.

The results supporting this hypothesis indicate that female physicians’ soft skills are more likely to be critiqued and that female physicians are much more likely to be associated with *disrespect*. We also present evidence that women may be penalized for lacking *candor* and *amicability* to a much greater degree than men. This may be attributed to failure to live up to a positive stereotype, as women are generally expected to be more open and personable, and female physicians in particular are expected to be more caring [46]. Furthermore, the physician role is one of authority, and research has extensively documented that women are punished for leadership styles that men are rewarded for [47,48].

### H2b: Soft Skills and Reviewer Gender

We hypothesized that female reviewers are more likely to reflect on their doctor’s soft skills. This hypothesis was supported by our study.

In positive reviews, women mentioned all positive soft skills with greater probability. However, the magnitude of these effects was diminished in negative reviews. These patterns suggest that women may be more inclined to value a physician’s soft skills. However, it also indicates that men may be more sensitive to a lack of positive soft skills than their presence.

We found that women were more likely to mention the negative soft skills, *indifference* and *disrespect*. This likely reflects the wealth of literature documenting the tendency for physicians to take women’s concerns less seriously and treat them with condescension [27,29,31].

### H3: Reviewer Gender and Physician Gender

We expected that female patients would report more negative experiences with male doctors. Our results support this hypothesis. Although there was no significant interaction effect of reviewer and physician gender on the probability of a negative experience, we found a significant interaction between reviewer and physician gender on the likelihood of reporting *disrespect*, which overwhelmingly occurs in negative reviews. Female reviewers apparently benefit from seeing female doctors, as they are less likely to mention *disrespect* when reviewing female physicians than when they review men. This compensating effect neutralized the overall greater association of *disrespect* with female reviewers, but not female doctors.

Given the literature and our previous finding that female physicians are subject to biased reviews, these results suggest that even women harbor bias against female physicians. However, this bias is considerably smaller among women than it is among men and is complicated by physician-gender/patient-gender preferences for different communication styles [32].

### Process and Physician Gender

We made no predictions regarding the relationship between *process* and soft skills or reviewer/physician gender. However, we found that both *amicability* and *disrespect* were highly correlated with *process*, suggesting that the ease of social interaction with staff is important to reviewers. Importantly, negative reviews of female physicians are considerably more likely to mention aspects of the clinical experience beyond experiences with the doctor.

The strong association between *process* and negative reviews of female doctors may reflect a tendency for patients to assess male doctors “on their own merits,” whereas women are more likely to be held accountable for poor process. This reflects a *bias against women* interpretation. A *realist* account might hypothesize that female doctors are more likely to work in clinics with less competent or accommodating staff.

### Limitations

This work is complementary to previous qualitative studies on the influence of gender on the doctor-patient relationship. We acknowledge that it is unclear whether gender differences reflect patient perception or the reality of physician behavior. For example, given the ample evidence in other contexts on bias against women, the high rate of negative reviews for female physicians likely reflects reviewer bias against physicians rather than genuine differences in treatment. However, our approach does not allow certainty in this regard. The Discussion section provides a more detailed explanation of the interpretation of gender differences in the context of the findings of previous studies. We also recognize that the data do not contain information related to patients’ health outcomes. Although the health outcome of each patient is not represented in our data, other studies have shown that reviews can reflect real health consequences [14]. Similarly, treatment noncompliance, unwarranted recalcitrance, and other patient characteristics beyond the reviewer’s narrative are not captured in the reviews.

In this paper and similar research, gender representation is reported as binary, which does not capture the full spectrum of gender or gendered interaction. Even though gender is likely to be interpreted in a binary fashion by most review readers [21], the doctor-patient relationship is more complex, and other data could offer more nuanced and richer perspectives. Furthermore, our work does not consider the intersection of gender and other identities, such as race [49,50].

As noted in the Methods section, the Google Places API limits data collection to 5 reviews per physician or practice. Google provides no documentation on how these reviews are chosen from all the reviews written. We acknowledge that the small sample may not be fully representative of a doctor or practice; however, our contribution is more focused on the biases within the reviews and not on the doctors themselves. We also note that the Google Places reviews are subject to selection biases. Demographics undoubtedly play a role in determining who writes physician reviews (eg, consider the high proportion of female reviewers in our data set). However, the data likely suffer selection biases, irrespective of demographic differences. For instance, it is probable that the U-shaped distribution of physician ratings is both a product of overall polarized attitudes and strong experiences providing greater motivation to write a review. It seems likely that other such selection biases were present in these data but were unknown to us as we performed our analysis.

### Summary and Recommendations

The increasing prevalence of online reviews of physicians affects both medical practices and patient choices. However, little is known about biases that may be present in these reviews or whether they reflect the real biases documented in doctor-patient interactions [27,28]. Conversely, most studies of gender bias in doctor-patient interactions to date have been limited to qualitative analyses, smaller-scale data sets, or specific medical conditions.

This study is the first to provide evidence that gender biases and other gender differences are observable at scale in physician reviews. We provide extensive evidence of differences in physician ratings and review content with respect to both physician and reviewer gender. Our statistical inference indicates that these differences are robust when controlling for possible confounding relationships and therefore are likely to reflect gender differences and biases in the patient-physician relationship.

It is difficult to disentangle which aspects of these gender differences may be attributed to review selection bias, gender bias, or gender behavioral variation. However, these patterns undeniably affect prospective patients as they peruse online reviews to select a doctor. Thus, it is important to consider how we might educate the public about the effects of gender bias on physician ratings and how online review systems could be improved to control for bias. We propose several concrete steps that could be taken to better support patients.

Review systems can draw attention to gender differences in reviews to aid prospective patients in building their own understanding of a physician’s potential gender biases. One way to do this is to organize reviews by gender. This could either be the default presentation or a special gender-separated view. Alternatively, reviewer gender might be indicated explicitly only in automatically generated summaries of physician reviews. Additionally, prospective patients might benefit from a panel that provides a sense of how a particular physician compares with other physicians on gender. For example, if there is a discrepancy between men’s and women’s ratings for a physician that differs greatly from the gender discrepancy of other similar physicians, a prospective patient might benefit from reading their reviews with this information in mind.

An online review system could also help to correct for gender differences that generally affect reviews. As female physicians receive many more negative reviews on average, a prospective patient might find it easier to select among physicians if ratings are adjusted to control for the physician’s gender or if ratings are reported relative to physicians of the same gender. On the other hand, online review systems could implement measures to reduce bias in the reviews. For instance, when writing a review and using a word that is commonly used to critique female physicians, the system could prompt the reviewer about gender stereotyping in word choice or alternative terms that are gender neutral. This might encourage a more balanced approach to review writing and help reviewers recognize their own biases. Alternatively, information can be solicited from the reviewer in such a way that greatly reduces the effects of gender stereotypes on performance evaluation [51].

These approaches would ideally lead to reviews that more accurately reflect the quality of care provided by physicians. Finally, this study draws attention to several important areas for future work. We advocate researchers adopt mixed methods approaches similar to the one presented here when pursuing quantitative analyses of text. Furthermore, this study raises questions specifically related to online review systems as objects of study in their own right. Little is known about how readers interpret online reviews, notably in the context of health care and gender. It also highlights the need to study how review systems can be designed to improve review accuracy and inform review readers and writers on gender bias in online reviews. We propose that experimental studies in review cognition and system design will be most fruitful to these ends.

## Appendix

Multimedia Appendix 1 Codebook.

## Abbreviations

NLP: natural language processing
OR: odds ratio

## References

[1] Rosenbaum L (2015). Scoring no goal-further adventures in transparency. N Engl J Med.

[2] Kadry B, Chu LF, Kadry B, Gammas D, Macario A (2011). Analysis of 4999 online physician ratings indicates that most patients give physicians a favorable rating. J Med Internet Res.

[3] Gao G, McCullough JS, Agarwal R, Jha AK (2010). Are Doctors Created Equal? An Investigation of Online Ratings by Patients. Proceedings of the Work- shop on Information Systems and Economics.

[4] Ellimoottil C, Hart A, Greco K, Quek ML, Farooq A (2013). Online reviews of 500 urologists. J Urol.

[5] López A, Detz A, Ratanawongsa N, Sarkar U (2012). What patients say about their doctors online: a qualitative content analysis. J Gen Intern Med.

[6] Hong YA, Liang C, Radcliff TA, Wigfall LT, Street RL (2019). What do patients say about doctors online? A systematic review of studies on patient online reviews. J Med Internet Res.

[7] Emmert M, Meier F, Pisch F, Sander U (2013). Physician choice making and characteristics associated with using physician-rating websites: cross-sectional study. J Med Internet Res.

[8] Jain S (2010). Googling ourselves--what physicians can learn from online rating sites. N Engl J Med.

[9] Lee S (2013). 'I hate my doctor': reputation, defamation, and physician-review websites. Health Matrix Clevel.

[10] Healthgrades.

[11] RateMDs.

[12] Vitals.

[13] Yelp.

[14] Paul MJ, Wallace BC, Dredze M (2014). What Affects Patient (Dis)satisfaction? Analyzing Online Doctor Ratings with a Joint Topic-Sentiment Model. AAAI Press Technical Reports.

[15] Wallace BC, Paul MJ, Sarkar U, Trikalinos TA, Dredze M (2014). A large-scale quantitative analysis of latent factors and sentiment in online doctor reviews. J Am Med Inform Assoc.

[16] Nwachukwu BU, Adjei J, Trehan SK, Chang B, Amoo-Achampong K, Nguyen JT, Taylor SA, McCormick F, Ranawat AS (2016). Rating a sports medicine surgeon's 'quality' in the modern era: an analysis of popular physician online rating websites. HSS J.

[17] Johnson RL, Roter D, Powe NR, Cooper LA (2004). Patient race/ethnicity and quality of patient-physician communication during medical visits. Am J Public Health.

[18] Mike M (2010). Doctor Sues Patients Over Bad Yelp Reviews. Techdirt.

[19] Upmark M, Borg K, Alexanderson K (2007). Gender differences in experiencing negative encounters with healthcare: a study of long-term sickness absentees. Scand J Public Health.

[20] Cohoon JM, Aspray W (2008). Women and Information Technology: Research on Underrepresentation.

[21] West C, Zimmerman DH (2009). Accounting for doing gender. Gend Soc.

[22] Richards C, Bouman WP, Seal L, Barker MJ, Nieder TO, T'Sjoen G (2016). Non-binary or genderqueer genders. Int Rev Psychiatry.

[23] Rojo D, Esteban CG, Lazar M (2005). The gender of power: the female style in labour organizations. Feminist Critical Discourse Analysis: Gender, Power and Ideology in Discourse.

[24] Schmader T, Whitehead J, Wysocki VH (2007). A linguistic comparison of letters of recommendation for male and female chemistry and biochemistry job applicants. Sex Roles.

[25] Madera JM, Hebl MR, Martin RC (2009). Gender and letters of recommendation for academia: agentic and communal differences. J Appl Psychol.

[26] MacNell L, Driscoll A, Hunt AN (2014). What’s in a name: exposing gender bias in student ratings of teaching. Innov High Educ.

[27] Elderkin-Thompson V, Waitzkin H (1999). Differences in clinical communication by gender. J Gen Intern Med.

[28] Schmittdiel J, Grumbach K, Selby JV, Quesenberry CP (2000). Effect of physician and patient gender concordance on patient satisfaction and preventive care practices. J Gen Intern Med.

[29] Street Jr RL (2002). Gender differences in health care provider-patient communication: are they due to style, stereotypes, or accommodation?. Patient Educ Couns.

[30] Halbert CH, Armstrong K, Gandy OH, Shaker L (2006). Racial differences in trust in health care providers. Arch Intern Med.

[31] (1993). National Survey of Women's Health. The Commonwealth Fund.

[32] Mast MS, Hall JA, Roter DL (2007). Disentangling physician sex and physician communication style: their effects on patient satisfaction in a virtual medical visit. Patient Educ Couns.

[33] Sandhu H, Adams A, Singleton L, Clark-Carter D, Kidd J (2009). The impact of gender dyads on doctor-patient communication: a systematic review. Patient Educ Couns.

[34] Mast MS (2004). Dominance and gender in the physician-patient interaction. J Mens Health Gend.

[35] Nelson LK (2017). Computational grounded theory: a methodological framework. Sociol Methods Res.

[36] Overview: Introducing the API. Google Developers.

[37] (2014). Prostate Cancer Rates by State. Centers for Disease Control and Prevention.

[38] Weinstein M TAMS Analyzer for Macintosh OS X. TAMS Analyzer - SourceForge.

[39] Elmas F SexMachine 0.1.1. The Python Package Index.

[40] Ladwig K, Marten-Mittag B, Formanek B, Dammann G (2000). Gender differences of symptom reporting and medical health care utilization in the German population. Eur J Epidemiol.

[41] Bertakis KD, Azari R, Helms LJ, Callahan EJ, Robbins JA (2000). Gender differences in the utilization of health care services. J Fam Pract.

[42] Feingold A (1994). Gender differences in personality: a meta-analysis. Psychol Bull.

[43] Costa Jr PT, Terracciano A, McCrae RR (2001). Gender differences in personality traits across cultures: robust and surprising findings. J Pers Soc Psychol.

[44] Miller AJ, Worthington EL, McDaniel MA (2008). Gender and forgiveness: a meta–analytic review and research agenda. J Soc Clin Psychol.

[45] Gayle BM, Preiss RW, Allen M (2002). A meta-analytic interpretation of intimate and nonintimate interpersonal conflict. Interpersonal Communication Research: Advances Through Meta-analysis.

[46] Fennema K, Meyer DL, Owen N (1990). Sex of physician: patients' preferences and stereotypes. J Fam Pract.

[47] Eagly AH, Mladinic A, Otto S (2016). Are women evaluated more favorably than men?: an analysis of attitudes, beliefs, and emotions. Psychol Women Q.

[48] Eagly AH, Karau SJ (2002). Role congruity theory of prejudice toward female leaders. Psychol Rev.

[49] Crenshaw K (1989). Demarginalizing the intersection of race and sex: a black feminist critique of antidiscrimination doctrine, feminist theory and antiracist politics. Univ Chic Leg Forum.

[50] Young IM, Allen DS (2011). Justice and the Politics of Difference.

[51] Bauer CC, Baltes BB (2002). Reducing the effects of gender stereotypes on performance evaluations. Sex Roles.

