# Supplementary material for: Gender, Soft Skills, and Patient Experience in Online Physician Reviews: A Large-Scale Text Analysis
Source: J Med Internet Res. 2020 Jul 30;22(7):e14455. doi: 10.2196/14455 (PMC7426798; doi:10.2196/14455)
Supplement: Multimedia Appendix 1 [file jmir_v22i7e14455_app1.pdf]

Terms used to identify review content themes. Terms given are the Porter stems used in the text-mining, e.g., "friendly" → "friendli", "dismissed/dismissive" → "dismiss". Numbers beneath each term indicate the percentage of reviews that mention the term. † indicates terms which were not stemmed. "Kind" (marked with an *) picked up "kindness" and "kindly", but the text was edited to prevent confusion with "kinds" and "kind of", which do not relate to amicability. Similar measures were taken to ensure "ignor" was not conflated with "ignorant" or "ignorance".

Candor: 14.40%

answer	explain	honest	straight	direct	to the point	candid	candor
6.59	6.23	1.08	0.86	0.73	0.62	0.09	0.01

Trust: 9.00%

comfort	trust	support	at ease†	safe	reassur	trustworthi
3.99	2.74	1.10	0.97	0.43	0.42	0.15

Investment: 40.86%

care	listen	thorough†	attent	compassion	respect	compass	valu	invest
28.77	9.32	4.72	3.05	2.75	2.31	1.08	0.43	0.11

Amicability: 24.47%

friendli	nice	kind*	personable†	smile	sweet	warm	gentl	funny†	humor
9.58	6.78	5.88	1.83	1.13	1.10	0.87	0.74	0.53	0.52

Indifference: 2.37%

cold	dismiss	ignor*	uncar	unwelcom	heartless	hopeless	indiffer	abandon	brushed off
0.667	0.58	0.55	0.35	0.06	0.06	0.06	0.05	0.05	0.04

didn’t/don’t matter†	brush off	uncommun
0.06	0.01	0.00

Disrespect: 8.26%

rude	disrespect	condescend	arrog	lie	unfriendli	liar	impati	curt	harass	cocki	snippi
6.73	0.64	0.49	0.48	0.29	0.26	0.16	0.14	0.08	0.07	0.04	0.03

Process: 49.40%

staff	wait	nurs	insur	receptionist	money	front desk	charg	cost	billing†	price	front offic
30.59	13.36	7.27	5.25	2.98	2.94	2.42	1.82	1.18	1.06	0.73	0.69

expens	administr	booking†
0.49	0.28	0.10
